# Clinical use of [TIMP-2]•[IGFBP7] biomarker testing to assess risk of acute kidney injury in critical care: guidance from an expert panel

**DOI:** 10.1186/s13054-019-2504-8

**Published:** 2019-06-20

**Authors:** Louis M. Guzzi, Tobias Bergler, Brian Binnall, Daniel T. Engelman, Lui Forni, Michael J. Germain, Eric Gluck, Ivan Göcze, Michael Joannidis, Jay L. Koyner, V. Seenu Reddy, Thomas Rimmelé, Claudio Ronco, Julien Textoris, Alexander Zarbock, John A. Kellum

**Affiliations:** 1 Florida Hospital, 601 E. Rollins Street, Orlando, FL 32803 USA; 2 University Hospital Regensburg, Franz-Josef-Strauß-Allee 11, 93053 Regensburg, Germany; 3 Baystate Medical Center, 759 Chestnut Street, Springfield, MA 01107 USA; 4 The Royal Surrey County Hospital NHS Foundation Trust, Egerton Rd, Guildford, Surrey GU2 7XX UK; 5 University of Surrey, 388 Stag Hill, Guildford, Surrey GU2 7XH UK; 6 Swedish Covenant Hospital, 5145 N California Ave, Chicago, IL 60625 USA; 7 Division of Intensive Care and Emergency Medicine, Department of Internal Medicine, Medical University of Innsbruck, Anichstraße 35, 6020 Innsbruck, Austria; 8 Section of Nephrology, Department of Medicine, University of Chicago, 5841 South Maryland Ave, Suite S-507, MC5100, Chicago, IL 60637 USA; 9 Tristar Centennial Medical Center, 2400 Patterson St #307, Nashville, TN 37203 USA; 10 Hospices Civils de Lyon, Edouard Herriot Hospital, 5 Place d’Arsonval, 69003 Lyon, France; 11 Department of Nephrology University of Padua, Padua Italy; San Bortolo Hospital, Vicenza, Italy; International Renal Research Institute Vicenza, Vicenza, Italy; 12 bioMérieux, 5 Place d’Arsonval, 69003 Lyon, France; 13 University Hospital Münster, Albert-Schweitzer Campus 1, Building A1, 48149 Münster, Germany; 14 The Center for Critical Care Nephrology, Department of Critical Care Medicine, University of Pittsburgh, 3347 Forbes Avenue, Suite 220, Pittsburgh, PA 15213 USA; 15 Critical Care Medicine, Clinical & Translational Science, and Bioengineering, Center for Critical Care Nephrology, 3347 Forbes Avenue, Suite 220, Pittsburgh, PA 15213 USA

**Keywords:** Biomarker testing, Acute kidney injury, Critical care, Expert panel, Protocols, Clinical guidelines, Tissue inhibitor of metalloproteinases-2, Insulin-like growth factor binding protein 7, Biomarker technology, Diagnosis

## Abstract

**Background:**

The first FDA-approved test to assess risk for acute kidney injury (AKI), [TIMP-2]•[IGFBP7], is clinically available in many parts of the world, including the USA and Europe. We sought to understand how the test is currently being used clinically.

**Methods:**

We invited a group of experts knowledgeable on the utility of this test for kidney injury to a panel discussion regarding the appropriate use of the test. Specifically, we wanted to identify which patients would be appropriate for testing, how the results are interpreted, and what actions would be taken based on the results of the test. We used a modified Delphi method to prioritize specific populations for testing and actions based on biomarker test results. No attempt was made to evaluate the evidence in support of various actions however.

**Results:**

Our results indicate that clinical experts have developed similar practice patterns for use of the [TIMP-2]•[IGFBP7] test in Europe and North America. Patients undergoing major surgery (both cardiac and non-cardiac), those who were hemodynamically unstable, or those with sepsis appear to be priority patient populations for testing kidney stress. It was agreed that, in patients who tested positive, management of potentially nephrotoxic drugs and fluids would be a priority. Patients who tested negative may be candidates for “fast-track” protocols.

**Conclusion:**

In the experience of our expert panel, biomarker testing has been a priority after major surgery, hemodynamic instability, or sepsis. Our panel members reported that a positive test prompts management of nephrotoxic drugs as well as fluids, while patients with negative results are considered to be excellent candidates for “fast-track” protocols.

**Electronic supplementary material:**

The online version of this article (10.1186/s13054-019-2504-8) contains supplementary material, which is available to authorized users.

## Introduction

**Fig. 1:**
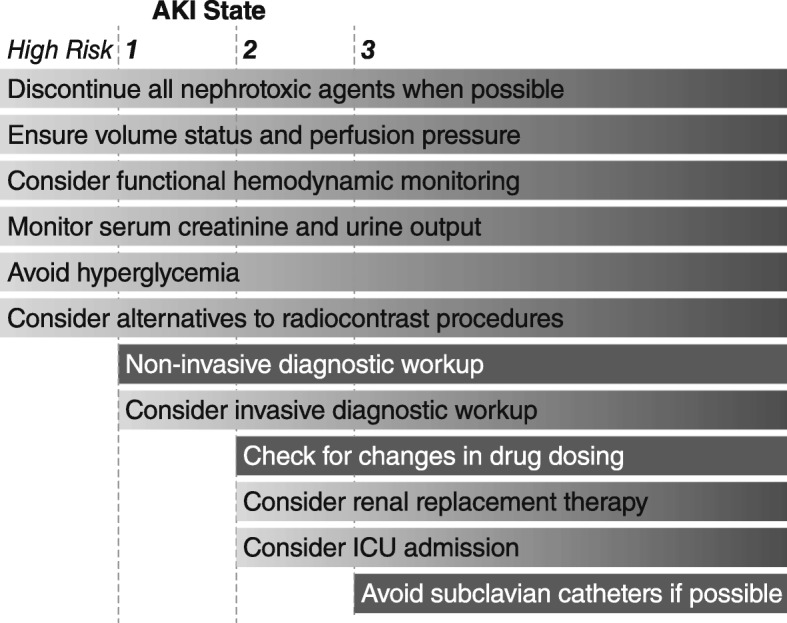
Stage-based management of acute kidney injury. Shading of boxes indicates priority of action; solid shading indicates actions that are equally appropriate at all stages whereas graded shading indicates increasing priority as intensity increases. AKI: acute kidney injury; ICU: intensive care unit. Source: Kidney Disease: Improving Global Outcomes (KDIGO) Acute Kidney Injury Work Group. KDIGO Clinical Practice Guideline for Acute Kidney Injury. *Kidney Inter* Suppl 2012, 2(1):1–138

Apart from renal replacement therapy, no direct treatment exists for acute kidney injury (AKI). Many researchers have argued that therapies could be translated from promising preclinical results if—and likely only if—patients could be identified in the very early stages of injury. Likewise, clinicians argue that early treatment—before irreversible injury occurs—would be much more likely to succeed than would potential interventions to reverse established AKI. The 2012 Kidney Disease: Improving Global Outcomes (KDIGO) clinical practice guideline [1] for AKI lists 12 actions; 6 measures should be implemented in patients at high risk for AKI, and only 6 are intended for patients with established AKI, whereas none are curative (Fig. 1).

To this end, researchers around the world have discovered biomarkers that can be detected in the blood or urine of patients before AKI is evident using standard clinical criteria (e.g., changes in serum creatinine and urine output). *N*-Acetyl-β-d-glucosaminidase (NAG), neutrophil gelatinase-associated lipocalin (NGAL), kidney injury molecule-1 (KIM-1), interleukin-18 (IL-18), and liver fatty acid-binding protein (L-FABP) were among the first candidates [1, 2]. More recently, tissue inhibitor of metalloproteinases-2 (TIMP-2) and insulin-like growth factor binding protein 7 (IGFBP7) have been added to the list [2]. With varying degrees of accuracy, these markers all provide information about the state of the kidney much earlier than do changes in function (i.e., serum creatinine).

In response to clinical need and their assessment of currently available evidence, some institutions have become early adopters of AKI biomarker technology. Because only one biomarker test is currently FDA approved for use in the USA, we focused on the NephroCheck® test (Astute Medical), which combines TIMP-2 and IGFBP7 ([TIMP-2]•[IGFBP7]). This test is also CE-marked (Conformité Européenne) and available in several European countries including France, Germany, Italy, Austria, Switzerland, the UK, and Spain. We sought to understand the following features of use to help determine how the test is currently being used: (1) Who are the target patients for [TIMP-2]•[IGFBP7] testing? (2) When are patients being tested (and retested)? (3) How are quantitative [TIMP-2]•[IGFBP7] test results being interpreted? (4) What actions are taken based on test results? To address these issues, a working group of clinical experts convened meetings to discuss their collective experience about the practicalities of implementing this test and to obtain group consensus regarding the most important clinical management actions to consider after obtaining a positive or negative test result. We recognize that many clinical actions are unproven, and we make no clinical recommendation on their use. Instead, our intent was to understand how the biomarker test was being used at sites that have adopted it.

## Methods

Invitations were sent to experts in critical care, nephrology, and surgery who had significant clinical experience with the biomarker. All invited experts were expected to have substantial experience using the test in clinical practice (i.e., not just in research studies). The recruitment of experts was based on personal knowledge of the investigators. Invitations were sent to 19 experts; 16 accepted. In 2018, two meetings were held, one in the USA and another in Europe, where experts described and discussed their clinical experiences with the use of [TIMP-2]•[IGFBP7] at their various institutions.

In advance of the meetings, invitees completed a questionnaire (Additional file 1: Table S1). The questionnaire was designed to collect information about different aspects of [TIMP-2]•[IGFBP7] testing, including factors that led to adoption at respondents’ respective institutions, specific items related to testing procedures, and interpretation of results. Collated results of the questionnaire were provided to group members before the meetings, so they could become familiar with all of the responses and be prepared to discuss the findings at each meeting.

In addition, participants were encouraged to provide their individual institution protocols and/or written instructions they had developed or with which they were familiar. These protocols were analyzed for common elements, and then rank-ordered by all participants (Table 1). The rank order of positive and negative [TIMP-2]•[IGFBP7] protocol actions and avoidances, respectively, may have had redundant bins, potentially skewing the “count” results. However, the goal of the protocol evaluation was to determine the best consensus actions using as many examples as possible rather than obtaining the most accurate “count” of protocol actions.

**Table 1:** [TIMP-2]•[IGFBP7] Protocol* Evaluation of existing clinical protocols: ranked order of actions and avoidances† by risk for AKI

Actions/avoidances		Actions/avoidances		Actions/avoidances	
Low risk (≤ 0.3)	Count	High risk (> 0.3, ≤ 2.0)	Count	Highest risk (> 2)	Count
Standard of care	13	No NSAIDs/ACE inhibitors/ARBs	20	Avoid aminoglycosides	6
Remove Foley	11	Keep/insert Foley	19	Renal ultrasound	5
Daily SCr	10	Hourly UO	19	Monitor SVV/Cardiac Index/SVO_2_ Q8–12	5
No HD monitoring	9	SCr Q8–12	19	Monitor fluid resuscitation	5
Recheck in 12 h if new insult	7	Avoid contrast	19	Maintenance fluids	5
Daily serum BUN	6	Consider/do renal consult	16	Send urine Na+, urea, creatinine	4
May use NSAIDs/ACE inhibitors	6	Recheck in 8–24 h	14	Check IVC compressibility with ultrasound	4
Diurese if signs of volume overload	6	Minimize/avoid nephrotoxins	13	Consider/use norepinephrine, epinephrine	4
SVO_2_ not monitored	5	Consider/use inotropes	11	Vasopressors	4
Mean hourly UO	4	Hold Lasix unless pulmonary edema	11	Dobutamine/Milrinone	4
Consider transfer out of ICU	4	Adjust medication dosing	10	Avoid multiple pressors	4
		Consider/do hemodynamic monitoring	9	Sensible fluids	4
		Adjust narcotics doses	9	Avoid and resolve hypervolemia (> 10% fluid gain)	4
		Consider colloids-only approach	9	Maintain SBP > 90	3
		Keep MAP > 65–80	8	Keep MAP ± 10% baseline	3
		Serum BUN Q12	7	Consider higher transfusion trigger	3
		Monitor SVO_2_ if history of abnormal liver function	7	PA catheter	3
		IVF expansion	7	Avoid piperacillin-tazobactam	3
		May use balanced fluid if CVP < 8 and hypovolemic	7	Low threshold for inotropes if Cardiac Index < 2, ScvO_2_ < 70, and/or LA increasing despite adequate MAP and volume expansion	3
		Pharmacy consult	7	Goal SVV < 14	2
		Urine Na, Cr, Eos ×1	6	Diuretics and fluids to be utilized only after determining fluid status and need with FloTrac, ultrasound, etc.	2
		Goal CI > 2.0–2.2	6	Assess fluid responsiveness	2
		Avoid vancomycin	6		

*25 [TIMP-2]•[IGFBP7] protocols evaluated. †Actions/avoidances included in ≥ 2 protocols. *ACE* angiotensin-converting enzyme, *AKI* acute kidney injury, *ARB* angiotensin-receptor blocker, *BUN* blood urea nitrogen, *Cr* creatinine, *CVP* central venous pressure, *Eos* eosinophils, *HD* hemodialysis, *ICU* intensive care unit, *IVC* inferior vena cava, *IVF* intravenous fluid, *MAP* mean arterial pressure, *Na* sodium, *NSAID* nonsteroidal anti-inflammatory drug, *PA* pulmonary artery, *SBP* systolic blood pressure, *SCr* serum creatinine, *SVO*_*2*_ venous oxygen saturation, *UO* urinary output, *SVV* stroke volume variation

At each meeting, the available protocols and questionnaire results were reviewed, and the panel agreed to discuss the four key questions related to the goals of the meeting:

i. Who are the target patients for [TIMP-2]•[IGFBP7] testing?
ii. When are patients being tested (and retested)?
iii. How are quantitative [TIMP-2]•[IGFBP7] test results being interpreted?
iv. What actions are taken based on test results?

Each question was then answered based on the clinical experience of the group along with information from the medical literature when available.

**Fig. 2:**
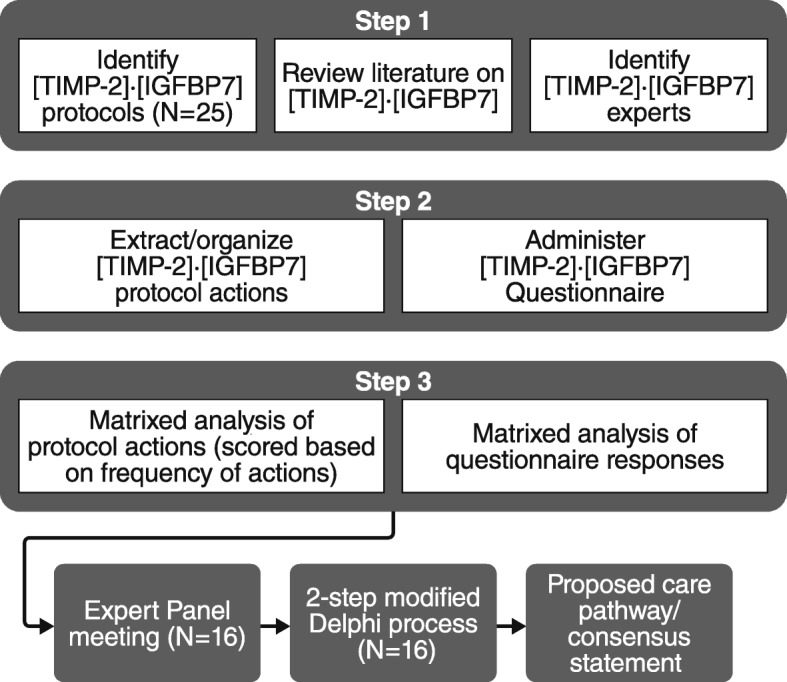
Developing proposed care pathway. A schematic representation of the steps involved before, during, and after the expert panel meetings

Next, we conducted a two-step modified Delphi process to ensure that our results were complete (step 1) and prioritized by the group (step 2). This process involved a single round of voting for each step. Ballots were anonymous to all but the senior author who tabulated the results. Figure 2 is a schematic representation of the steps involved before, during, and after the expert panel meetings.

## Results

### Q1. Who are the target patients for [TIMP-2]•[IGFBP7] testing?

The expert panel agreed on key candidates for biomarker testing based on their direct experience, with the caveat that this list might not be exhaustive. Proposed target populations are specified in rank order in Table 2. Postoperative cardiac or major vascular surgery was the most strongly supported followed by shock/hemodynamically unstable patients regardless of the cause and sepsis (with or without shock). Further down on the list, but still with strong support, were postoperative non-cardiovascular major surgery, cardiac arrest/extracorporeal membrane oxygenation, and patients with persistent oliguria after resuscitation. Additional populations suggested by the group are shown in Table 2.

**Table 2:** Proposed target patient populations for [TIMP-2]•[IGFBP7] testing

Tier 1	
• Postoperative cardiovascular surgery	
• Shock/Hemodynamically unstable	
• Sepsis	
• Postoperative major non-cardiovascular surgery	
• Cardiac arrest, extracorporeal membrane oxygenation	
• Oliguria after acute resuscitation	
Tier 2	
• Severe trauma (Injury Severity Score > 15)	
• Acute illness/decompensation	
• Elevated serum creatinine and no baseline	
• Decompensated heart failure	
• Acute respiratory distress syndrome/hypoxic respiratory failure	
• Burn patients with total body surface area > 30%	
• Anyone being seen by a rapid response team	
• Solid organ (liver, heart, lung, kidney) transplants	
• Receiving any nephrotoxic medications	
• Any unplanned intensive care unit admission	
• Suspected (impending) stage 2/3 acute kidney injury	
• Volume depleted	
• End-stage liver disease with early acute kidney injury (± hepatorenal syndrome)	
• Post-urologic procedure (e.g., partial / radical nephrectomy or cystectomy)	

Populations are listed in order of priority. Priorities assigned to the top three populations were highest among all participants (scores > 35 out of a possible 48). The second tier was also highly ranked (> 20). The remaining populations received lower priority rankings (10–20)

### Q2. When are patients being tested (and retested)?

**Fig. 3:**
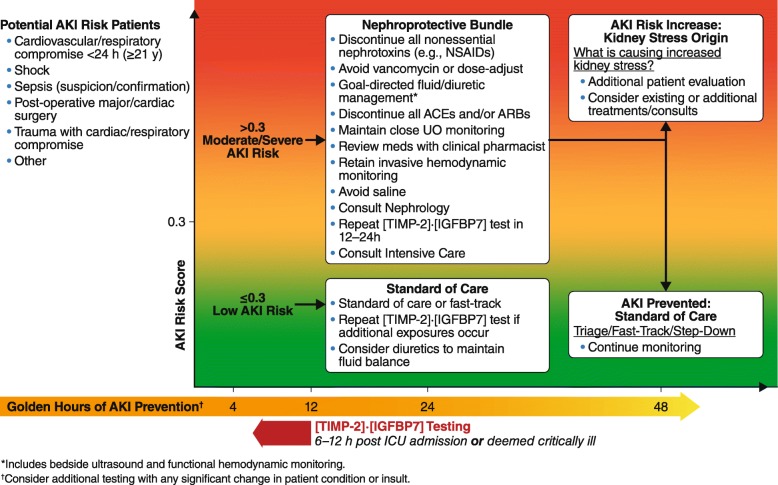
Protocol for [TIMP-2]•[IGFBP7] testing. ACEs, angiotensin-converting enzymes; AKI, acute kidney injury; ARBs, angiotensin-receptor blockers; ICU, intensive care unit; NSAIDS, nonsteroidal anti-inflammatory drugs; UO, urinary output

In general, [TIMP-2]•[IGFBP7] testing has been ordered for patients when the kidney is under threat for any reason—when something creates a toxic event in the kidney, when there is a question of secondary nephrotoxicity, or at any time a significant change in status has occurred that might result in kidney injury. Most users found that [TIMP-2]•[IGFBP7] testing is particularly useful within the first 72 h of ICU admission. Therefore, [TIMP-2]•[IGFBP7] testing is most often being ordered in patients at risk for AKI, including those who are hemodynamically unstable, in respiratory failure, or exhibiting Stage 1 AKI (Fig. 3). Specific clinical scenarios (e.g., cardiac surgery, sepsis) were identified where appropriate testing might differ.

### Q3. How are quantitative [TIMP-2]•[IGFBP7] test results being interpreted?

The FDA has approved the cutoff threshold of > 0.3, which ensures that > 92% of all stages 2/3 AKI events over the following 12 h are predicted [3–5]. A low risk for AKI is defined as a test result ≤ 0.3. Moderate risk is defined as having a result of > 0.3 and ≤ 2.0; high risk is associated with a result > 2.0. Thus, as test results increase, so does the level of kidney stress and the risk of AKI. If the result is > 0.3, there is a “risk” and any potential damage needs to be averted [6, 7]. All of the protocols reviewed used the 0.3 cutoff to separate low from moderate-high risk. A few protocols also specified actions related to the 2.0 cutoff, which generally related to increasing intensity of actions triggered by the 0.3 cutoff (e.g., more frequent monitoring of serum creatinine).

### Q4. What actions are taken based on test results?

Meeting participants discussed and came to consensus regarding recommended actions (both positive and negative—i.e., things to do as well as things not to do) when the [TIMP-2]•[IGFBP7] test is positive. A complete list of rank-ordered actions after a positive or negative [TIMP-2]•[IGFBP7] test is provided in Table 3. Actions chosen by the group were often found in the KDIGO guideline, as well as in more recent recommendations for prevention of AKI [1, 8]. The highest priority actions fell into two domains, management of nephrotoxins and fluids. Top priority was given to “discontinue all nonessential potential nephrotoxins (e.g., NSAIDs [nonsteroidal anti-inflammatory drugs])”; avoiding vancomycin (or dose adjusting) especially combinations of vancomycin with aminoglycosides or piperacillin tazobactam, and discontinuing angiotensin-converting enzyme (ACE) inhibitors or angiotensin-receptor blockers (ARBs) were ranked second and fourth. Goal-directed fluid management (e.g., bedside ultrasound and functional hemodynamic monitoring) was ranked third, and retaining invasive hemodynamic monitoring was ranked seventh. A negative [TIMP-2]•[IGFBP7] test result was seen as just as informative and actionable as a positive test, because low-risk patients may benefit from many of the treatments best avoided in high-risk patients (e.g., NSAIDs). There was strong consensus that patients with a negative test were good candidates for “fast-track” protocols and rapid de-escalation of monitoring (e.g., removal of arterial lines, indwelling urinary catheters). Of note, five authors expressed that they had originally doubted that the information provided by the test would change clinical practice. Only after using the test in several patients were they convinced.

**Table 3:** Consensus statements for potential actions after positive and negative [TIMP-2]•[IGFBP7] testing

Positive test (> 0.3)	Negative test (≤ 0.3)
• Discontinue all nonessential nephrotoxins (e.g., NSAIDs) • Avoid vancomycin or dose adjust • Goal-directed fluid/diuretic management*	• Standard of care or fast-track • Repeat [TIMP-2]•[IGFBP7] test if additional exposures occur • Consider diuretics to maintain fluid balance
• Discontinue all ACE inhibitors and/or ARBs • Maintain close UO monitoring • Review meds with clinical pharmacist • Retain invasive hemodynamic monitoring • Avoid saline	
• Consult nephrology • Repeat [TIMP-2]•[IGFBP7] test in 12–24 h • Consult intensive care	

*Includes bedside ultrasound, and functional hemodynamic monitoring

Actions are listed in order of priority. High priority (> 30 out of a possible 48) was assigned to the top 5 actions. Actions that received a score < 12 (equivalent to low priority by all participants and more than 25% of participants not supporting at all) were removed from the list

## Discussion

Clinical experts from the Europe and North America had very similar experiences with the clinical use of the test. Types of patients being tested and the types of actions based on the test result were similar. More variation was seen in terms of when to test. Some areas emphasized by the participants warrant further discussion.

### Which patients to test

#### All postoperative cardiac surgery patients

The Society of Thoracic Surgeons’ Adult Cardiac Surgery Database contains more than 6.5 million cardiac surgery procedure records and currently has approximately 3800 participating physicians [9]. After cardiovascular surgery, between 5 and 10% of patients develop kidney failure, defined by a threefold increase in serum creatinine from baseline or increase to a creatinine > 4 mg/dL (which would be equivalent to KDIGO criteria stage 3 AKI by creatinine [1]). Rates of AKI by full KDIGO criteria are much higher, approaching 2 in 3 patients [10]. Specific considerations for cardiac surgery patients include the common practice of reducing circulating fluid volume on cardiopulmonary bypass (hemoconcentration) often resulting in postoperative oliguria. Current medical literature has demonstrated that post-cardiac surgery patients, identified as high risk by biomarker testing, and randomized to a KDIGO treatment had up to a 34% reduction in stage 2/3 AKI compared with those patients randomized to standard of care [7]. Similar results were reported for non-cardiac major surgery. [11]

#### Patients with shock or hemodynamic instability regardless of the cause

Patients with shock, including hypovolemic, distributive/septic, and cardiogenic, as well as patients with acute decompensated heart failure, are at high risk for AKI.

#### Patients with sepsis

Sepsis is the most common cause of AKI in the ICU [12]. Patients with sepsis, particularly those with septic shock also have more severe AKI—in a recent study, 44% of septic shock patients developed stage 2–3 AKI [13].

#### Unplanned admission to the ICU

Patients admitted to the ICU from the ward or emergency department are often at high-risk for AKI, especially if they are hemodynamically unstable or septic. The initial studies validating [TIMP-2]•[IGFBP7] enrolled patients with cardiovascular or respiratory failure going to the ICU [2]. For practical purposes, this includes most unplanned admissions and some planned postoperative admissions (e.g., cardiac surgery). Patients assessed for possible ICU admission by a rapid response team are also at high risk, and [TIMP-2]•[IGFBP7] testing is currently being used by some institutions to help evaluate and triage these patients.

### When to test

For cardiac surgery patients, most measured [TIMP-2]•[IGFBP7] within 4 h post-surgery. Performing the test at a few different postoperative timepoints is helpful to identify the most appropriate testing time for a particular program given inherent differences in care across institutions. Studies have reported a variety of results in this regard. In several studies [14–16], [TIMP-2]•[IGFBP7] detected elevations at 4 h; in another study, elevations were not detected until the day after surgery [17]. A recent study [18], with the most granular time-course published to date, shows bimodal elevations of [TIMP-2]•[IGFBP7] with the first peak occurring intraoperatively and the second 6 h after ICU admission in patients who developed stages 2/3 AKI. The authors postulate that the first peak indicates kidney stress caused during the surgery, while the second peak may indicate kidney stress caused during early postoperative care. Measurement at both times resulted in the best predictive ability, as would be expected for two independent episodes of stress.

For patients in shock, [TIMP-2]•[IGFBP7] testing is ordered as early as possible during patient evaluation. Interestingly, there is emerging evidence in septic shock that the post-resuscitation test results may be most predictive [19]. However, it also has been noted that when test results improve (levels decrease) with resuscitation, outcomes are better. There is therefore the hypothesis that [TIMP-2]•[IGFBP7] testing might ultimately be proven useful as a tool to monitor resuscitation efficacy. Establishing clinical utility for this indication will require studies that compare a biomarker-guided approach to a standard approach. Such studies are currently lacking.

### What actions to take and how to integrate the technology into practice

Management of potential nephrotoxic medications was the top priority in patients with positive test results. The clinical panel participants agreed that all nonessential nephrotoxic medications should be avoided. The combination of vancomycin and piperacillin-tazobactam, in particular, has been noted to significantly increase risk for AKI [20]. If vancomycin (or an aminoglycoside) is used, it should be dosed strictly by levels, and its duration of use should be as limited as possible. If a pharmacist is not already part of the critical care team, consultation may be appropriate. Likewise, NSAIDs and ACE inhibitors/ARBs should be avoided in the early postoperative period.

A second category of high-priority actions involved fluid management. Participants noted that patients with a positive test result are at risk for fluid overload but also might be volume depleted. There was strong consensus therefore that a “goal-directed” approach to fluid/diuretic management was essential. Two examples of such an approach were published, one in 2017 in cardiac surgery patients [7] and one in 2018 in non-cardiac surgery patients [11]. In the first study, biomarker-positive patients were randomized to receive a care bundle that included a hemodynamic management algorithm based on mean arterial pressure and stroke volume variation. AKI was significantly reduced with the intervention compared to controls (55.1 vs. 71.7%; ARR 16.6% (95% CI 5.5–27.9%); *p* = 0.004). Rates of moderate to severe AKI were also significantly reduced by the intervention compared to controls (41/138 (29.7%) vs 62/138 (44.9%); *p* = 0.009; OR, 0.518 (95% CI, 0.316–0.851); ARR, 15.2% (95% CI, 4.0–26.5%)). The intervention resulted in significantly improved hemodynamics (*p* < 0.05) as well as less hyperglycemia (*p* < 0.001) and use of ACEi/ARBs (*p* < 0.001) compared to controls. The total administered volume was not different between the two groups, but the distribution of fluid was different, with patients in the intervention group receiving significantly less volume during the last 3 h of the intervention period (*p* = 0.024). However, there were no differences in rates of renal replacement therapy between intervention and control either within 72 h (7.2% vs. 5.1%, *p* = 0.45), during hospitalization (10.1% vs. 6.5%, *p* = 0.28), or at 30 days (3.1% vs. 2.3%. *p* = 0.72). Neither were there differences in mortality or persistent renal dysfunction at 30, 60, or 90 days.

In the second study [11], a similar care bundle including early optimization of fluid status and maintenance of perfusion pressure, was applied to non-cardiac major surgery patients after testing positive for the biomarker. Overall AKI rates were not statistically different between groups (19/60 (31.7%) in the intervention group vs. 29/61 (47.5%) in the standard care group, *p* = 0.076). However, rates of moderate and severe AKI, a secondary endpoint, were reduced with the intervention (4/60 (6.7%) vs. 12/61 (19.7%), *p* = 0.04), as were lengths of ICU stay (median difference 1 day, *p* = 0.035) and hospital stay (median difference 5 days, *p* = 0.04). There were no significant differences regarding renal replacement therapy, in-hospital mortality, or major kidney events at hospital discharge. Interestingly, 48-h cumulative balance was not statistically different (2567 ml (1617–3706) vs. 3207 ml (2015–4486), *p* = 0.085) but favored lower volumes in the intervention group. This last finding brings to attention the fact that “optimization” of fluid status for AKI patients does not mean “give fluid” and frequently results in less fluid.

As with any new technology, there are potential barriers to adoption. The value proposition for new technology involves both potential benefits as well as costs. Although a cost-effectiveness analysis for the test is beyond the scope of this report, it is notable that AKI is extremely expensive—estimates put the cost at more than 19,000 USD [21] to as much as 39,000 USD per case [22]. By comparison, the test itself retails for approximately 100 USD per determination. However, the test will not be useful in all patients, and protocols should define appropriate lines of communication to ensure that the [TIMP-2]•[IGFBP7] test is ordered for the appropriate patients. Given that [TIMP-2]•[IGFBP7] provides an early warning of kidney stress, the test results are most valuable when reported promptly. Therefore, the [TIMP-2]•[IGFBP7] should be ordered as a “stat” (i.e., as soon as possible) test so that results are available as quickly as possible. Most participants reported a 1-h turn around by their clinical lab. By instituting an AKI biomarker protocol, hospitals have the opportunity to develop and test metrics that can enhance quality improvement initiatives.

As part of [TIMP-2]•[IGFBP7] test protocol integration, the inclusion of information technology and the electronic health record (EHR)/electronic medical record (EMR) system is imperative to ensure that test reporting is online, and the test is used consistently. The test may become a part of cardiopulmonary bypass protocols and can be added as an EHR order. It is helpful to provide sample protocols and data to demonstrate what is needed. If [TIMP-2]•[IGFBP7] testing is effectively integrated into these systems, it also may be beneficial to expand testing into other hospital systems and networks. For example, [TIMP-2]•[IGFBP7] testing could work well in other ICUs, for all patients admitted with shock, the emergency department, and/or trauma unit, as well as operating rooms.

Despite its brief history, dozens of studies have evaluated the diagnostic value of [TIMP-2]•[IGFBP7] for AKI, in various settings (e.g., cardiac surgery, ICU, emergency department/trauma), different patient populations (e.g., KDIGO criteria, elderly, high-risk surgeries), and measurement criteria (e.g., thresholds, sampling times). Detailed discussion of these studies is beyond the scope of this report, but several systematic reviews are available [23–27]. Overall, [TIMP-2]•[IGFBP7] is accurate in identifying patients at risk for AKI. However, to our knowledge, only two studies, thus far, have attempted to evaluate whether use of the test alters the clinical course of AKI [7, 11]. Thus, future research is warranted to better understand how treatment protocols based on [TIMP-2]•[IGFBP7] results can improve outcomes.

## Conclusions

Clinical experts have developed very similar practice patterns for use of the [TIMP-2]•[IGFBP7] test on both sides of the Atlantic. Strong consensus was achieved for whom to test, how to interpret a test result, and for the actions to take based on test results. Importantly, many actions listed in Table 1, though common in clinical practice, are not supported by scientific evidence but rather reflect the clinical judgment of the authors. Less consensus was present on when to test, but this also reflected different patient populations. In general, testing needs to occur early but only after a potential inciting event has occurred. Patients undergoing major surgery (cardiac or non-cardiac), those with hemodynamic instability, or those with sepsis were believed to be the top priority patient populations for the biomarker test. Top actions for positive tests involve management of nephrotoxic drugs as well as fluids. Patients testing negative were considered to be excellent candidates for “fast-track” protocols.

## Additional file

Additional file 1: **Table S1.** [TIMP-2]•[IGFBP7] User Group 1 Questionnaire. (DOCX 17 kb)

## Data Availability

Data sharing is not applicable to this article as no datasets were generated or analyzed during the current study.

## Abbreviations

ACE: Angiotensin-converting enzyme
AKI: Acute kidney injury
ARB: Angiotensin-receptor blocker
BUN: Blood urea nitrogen
CI: Confidence Interval
Cr: Creatinine
CVP: Central venous pressure
EHR: Electronic health record
EMR: Electronic medical record
Eos: Eosinophils
HD: Hemodialysis
ICU: Intensive care unit
IGFBP7: Insulin-like growth factor binding protein 7
IL-18: Interleukin-18
IVC: Inferior vena cava
KDIGO: Kidney Disease: Improving Global Outcomes
KIM-1: Kidney injury molecule-1
L-FABP: Liver fatty acid-binding protein
MAP: Mean arterial pressure
Na: Sodium
NAG: *N*-Acetyl-β-d-glucosaminidase
NGAL: Neutrophil gelatinase-associated lipocalin
NSAID: Nonsteroidal anti-inflammatory drug
SBP: Systolic blood pressure
SCr: Serum creatinine
SVO_2_: Venous oxygen saturation
TIMP-2: Tissue inhibitor of metalloproteinases-2
UO: Urinary output
