# Supplementary material for: Clinical use of [TIMP-2]•[IGFBP7] biomarker testing to assess risk of acute kidney injury in critical care: guidance from an expert panel
Source: Crit Care. 2019 Jun 20;23:225. doi: 10.1186/s13054-019-2504-8 (PMC6585126; doi:10.1186/s13054-019-2504-8)
Supplement: Supplementary file 1 — Table S1. [TIMP-2]•[IGFBP7] User Group 1 Questionnaire. (DOCX 17 kb) [file 13054_2019_2504_MOESM1_ESM.docx]

**Table S1.** [TIMP-2]•[IGFBP7] User Group 1 Questionnaire

| **Adoption of [TIMP-2]•[IGFBP7] testing** |
| --- |
| - What were the most important considerations that convinced you that trying [TIMP-2]•[IGFBP7] testing was a good idea? |
| - What were your biggest doubts, questions, or sources of skepticism? |
| - What do you know now that you wish you knew when you started using [TIMP-2]•[IGFBP7] testing? |
| **[TIMP-2]•[IGFBP7] testing procedures: Who? When? Where?** |
| - What patients are appropriate candidates for the [TIMP-2]•[IGFBP7] test? |
| - When should [TIMP-2]•[IGFBP7] testing be ordered? |
| - What triggers additional [TIMP-2]•[IGFBP7] testing? |
| **Interpreting [TIMP-2]•[IGFBP7] test results** |
| - How ought one interpret the [TIMP-2]•[IGFBP7] score (including initial test and follow-up test scores?) |
| - If the test result is positive (above the FDA cut-off of 0.3), what are the next steps in patient care or specific checklist of actions or interventional considerations that should be included in a general protocol? |
| - Assuming positive, what method, technique or device are you using to obtain objective data about volume status? |
| - If the test result is negative (at or below the FDA cutoff of 0.3), what are the next steps in patient care or specific checklist of actions or interventional considerations that could be more aggressively used in a general protocol? |
| - Does the negative predictive value help in decision making? |
| - Should other cutoffs (test scores) than 0.3 be used to indicate differing interventional approaches? |
| - Are there any additional important questions to answer for new users of NephroCheck? |
| - What are the most important questions open for you at this time? |
| **Institutional considerations/strategies for adoption of [TIMP-2]•[IGFBP7] testing** |
| - What pitfalls/roadblocks did you have to overcome during hospital approval/implementation? |
| - Establishing how the hospital “wins” is important, as is coordinating the various functional (and budgetary) silos into an integrated process. What clinical and/or economic data points, information, or processes were important for convincing your hospital to proceed with [TIMP-2]•[IGFBP7] testing implementation? |
| - Are you incented or compensated on metrics or key performance indicators? Do you see [TIMP-2]•[IGFBP7] and AKI as being a metric/key performance indicator that can be incorporated into incentives and compensation? |
| - Do you believe that including [TIMP-2]•[IGFBP7] testing into the EHR/EMR system is important for consistent test usage? |
| - To what extent is your EHR built to facilitate [TIMP-2]•[IGFBP7]-test ordering, interpret results, and determine interventions? And what EHR do you use? |
| - What advice, process templates, or best practices could you share with new users to enable timely and successful IT integration of their [TIMP-2]•[IGFBP7] testing project? |
| - What additional advice would you give inexperienced users in their hospital implementation endeavors? |
| **Expanding use of [TIMP-2]•[IGFBP7] testing** |
| - Do you see NephroCheck protocols being applicable for patients in the emergency department or trauma settings? |
| - How do you envision expanded use within your hospital institution and/or network? |
| - Pooling and/or publishing collective user data may be beyond the practical scope of the [TIMP-2]•[IGFBP7] testing User Group I, but could be the subject for future user groups or a registry. What data have you collected and what have you demonstrated with regard to patient outcomes, cost, or other metrics? |
| - If rigorous data analysis is beyond the scope of this User Group, should data collation be a scope for a [TIMP-2]•[IGFBP7] testing User Group II, and if so, what data might be collected? |
